# Evaluation of a Web-Based Self-Help Intervention for Patients With Generalized Anxiety Disorder: Protocol for a Randomized Controlled Trial

**DOI:** 10.2196/41440

**Published:** 2023-07-26

**Authors:** Julian Rubel, Jannis Quest, Luise Pruessner, Christina Timm, Steffen Hartmann, Sven Barnow, Lisa Rittmeyer, David Rosenbaum, Christopher Lalk

**Affiliations:** 1 Faculty of Psychology and Sport Science Justus-Liebig-University Giessen Gießen Germany; 2 Faculty of Behavioral and Empirical Cultural Studies Heidelberg University Heidelberg Germany; 3 Clinic and Polyclinic for Psychiatry and Psychotherapy University Hospital Tuebingen Tuebingen Germany

**Keywords:** generalized anxiety disorder, GAD, online self-help, randomized controlled trial, RCT, self-help, guided, anxiety, online intervention, mental health, mental illness, mental disorder, psychotherapy, internet-based, internet intervention, web-based

## Abstract

**Background:**

Generalized anxiety disorder (GAD) is a highly prevalent and severely distressing condition that can lead to functional impairments and is considered one of the most difficult anxiety disorders to treat. Following new technological developments, a highly structured cognitive behavioral therapy (CBT) approach that has already shown success in face-to-face psychotherapy can be implemented: internet-delivered CBT (iCBT). There is now evidence for the efficacy of both guided and unguided iCBT interventions for GAD regarding symptom reduction.

**Objective:**

To establish the usefulness of such interventions, we plan to evaluate the efficacy of a web-based self-help program (Selfapy) for GAD in a relatively large sample. We aim to assess effects beyond symptom reduction, including effects on well-being, functioning, and mental health literacy, as well as the effect on health care burden, while testing the intervention in conditions comparable to routine care.

**Methods:**

Patients (n=156) who have been diagnosed with GAD, are aged between 18 and 65 years, have internet access, and have sufficient German language skills will be recruited for this study. The intervention group (n=78) will receive access to the 12-week self-help web-based program Selfapy. The waitlist control group (n=78) will receive no intervention in the context of the study. However, both groups will be allowed to access further health care services (eg, psychotherapy, medication), reflecting current routine care in Germany. Outcome measures will be assessed at baseline (T1) and 6 weeks (T2) and 12 weeks (T3) after the start of the intervention. The primary outcome will be generalized anxiety symptoms and quality of life at T3. Additional outcomes include depression, work capacity, therapy-related expenses and burdens, health literacy, and negative effects.

**Results:**

By May 2023, all participants had finished the trial and the report was being prepared for publication.

**Conclusions:**

Web-based interventions may be an important addition to the German health care system to reduce barriers to treatment access. Further, they may prove cost-effective for the treatment of GAD.

**Trial Registration:**

Deutsches Register Klinischer Studien DRKS00023799; https://tinyurl.com/22bds38x

**International Registered Report Identifier (IRRID):**

DERR1-10.2196/41440

## Introduction

### Background

Generalized anxiety disorder (GAD) is characterized by generalized and persistent excessive worry about various aspects of the past, present, or future without being confined to specific environmental conditions [1]. GAD can lead to severe distress and limitations in the daily lives of those affected [2] and is often associated with comorbid depressive disorders [3]. Subsequently, GAD poses an economic challenge to the health care system [4,5]. Lifetime prevalence is estimated at 6.2% [6].

GAD can be successfully treated by pharmacotherapy or psychotherapy [7]. Still, as individuals affected by GAD show lower response rates to therapies than other anxiety disorders, it is considered one of the most challenging anxiety disorders to treat [8]. One reason may be that individuals with GAD wait on average about a decade until they begin treatment [9]. Therefore, many cases remain undiagnosed and untreated, especially in the context of long waiting times and limited resources [9]. Following technological advancements, technology-based treatment alternatives have been increasingly developed over the past years to address the aforementioned problems [10]. Cognitive behavioral therapy (CBT) is especially suited for the transfer to web-based interventions due to its highly structured, directive, and standardized nature, as well as its emphasis on psychoeducation and homework [11]. In addition to being independent of limited resources, internet-delivered CBT (iCBT) also provides easier access for those who reject traditional forms of therapy due to stigma or other reasons [12].

Systematic reviews and meta-analyses [13] suggest that the use of iCBT programs for GAD compared to inactive control groups was significantly more effective and had moderate to large effect sizes (Hedges *g*=0.79) in reducing GAD symptoms. Further, iCBT programs for GAD achieved comparable effects to face-to-face CBT while the time required for each patient was 7.8 to 13 times less [14-17]. Of particular note is the benefit of iCBT to the health care system, as it may be a more cost-effective treatment alternative for GAD [18] since it can help reduce the waiting time for outpatient therapy, which averages 5 months in Germany [19] and has increased since the beginning of the COVID-19 pandemic [20].

While most of the literature focuses on guided forms of iCBT with therapeutic support, minimally guided, self-directed iCBT programs also show large effect sizes in the treatment of GAD (eg, Cohen *d*=2.43 [21]). Dear et al [22] found no significant differences between guided and unguided iCBT in terms of symptom reduction, completion rates, and satisfaction in the treatment of GAD. Moreover, first evidence suggests that characteristics of the guidance, such as the frequency of supportive monitoring [23], type of reminder (eg, phone, email), and the experience or training of the support person [24], do not have a significant influence on GAD symptom reduction. Nevertheless, it is important to take steps to promote adherence and minimize the typically high dropout rates of web-based, particularly self-directed, interventions [25] (eg, motivational content).

### Objective

Taken together, minimally guided web-based interventions provide a low-threshold method of access to mental health care and may be particularly helpful for GAD patients who often do not seek help [9]. We aim to establish further evidence for a web-based self-help program in a setting comparable to routine care. For this purpose, a comparison is made with a wait-list control group within a randomized controlled trial framework. Further, we want to address outcomes beyond symptom reduction, such as functioning in both daily life and the workplace, as well as mental health literacy and the health care burden.

## Methods

### Participants

Recruitment is carried out through newsletters and social media advertising drawing attention to Selfapy. Interested individuals can register for participation on the web, and after a successful video call–based screening procedure they will be contacted by the study center to schedule a diagnostic appointment.

Video calls will be conducted with all subjects, during which eligibility will be evaluated based on a diagnostic interview, the Diagnostic Interview for Mental Disorders–Open Access (DIPS-OA) [26]. Trained interviewers with at least a master’s degree (or equivalent) in psychology focusing on clinical psychology will conduct all interviews. The interviewers are trained at the Justus Liebig University in Giessen, Germany. Furthermore, there is close supervision on questions of diagnostics, as well as the inclusion and exclusion criteria, by a psychotherapist licensed in CBT.

Eligible subjects (1) are aged between 18 and 65 years, (2) have sufficient knowledge of the German language, (3) have uninterrupted internet access, (4) provide electronic informed consent to participate in the study, and (5) currently meet the diagnostic criteria according to the Diagnostic and Statistical Manual of Mental Disorders [1] for a diagnosis of GAD (International Classification of Diseases, 10th Revision code F41.1).

Subjects will be excluded if they do not fulfill any of the inclusion criteria or meet any of the following exclusion criteria: (1) past or current diagnosis of bipolar disorder, (2) past or current diagnosis of psychotic disorder, (3) current diagnosis of substance dependence, (4) current diagnosis of a severe major depressive episode, and (5) acute suicidality. A primary diagnosis of a disorder other than GAD is not an exclusion criterion, as we want to represent routine care. However, substance dependence, bipolar disorder, or psychotic illness are exclusion criteria because they conflict with the implementation of the program. Subjects who do not meet our inclusion criteria due to severity of illness are encouraged to seek professional help. Adequate language skills will be determined during the initial interview.

### Study Design

**Figure 1:**
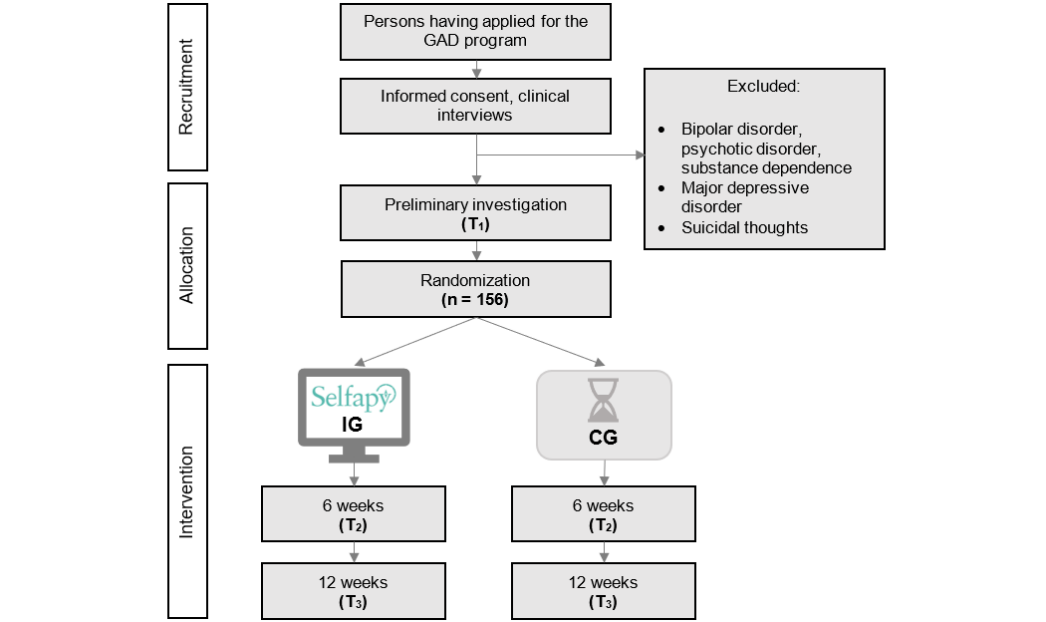
Flowchart of the study design for intervention and control groups. GAD: generalized anxiety disorder; CG: control group; IG: intervention group.

A 2-arm randomized controlled trial is being conducted to test the efficacy of the minimally guided web-based program Selfapy for GAD. The web-based course is defined as minimally guided as it is completed independently by the participant. However, as part of the patient safety concept, a psychologist monitors the participant’s progress to support the patient and respond to adverse events, such as suicidality. The participants can ask a psychologist questions about the program via an integrated messaging function. Figure 1 shows the schematic study design for patients in the intervention group and control group. After a structured diagnostic interview (DIPS-OA [26]) to clarify the inclusion and exclusion criteria, eligible patients will receive the first questionnaire and subsequently will be randomly assigned to the intervention group or control group. Patients in the intervention group will be able to use Selfapy immediately after randomization, while the control group will only have access to Selfapy after a waiting period of 12 weeks. Interim and final evaluations will occur 6 weeks (T2) and 12 weeks (T3) after the entry survey.

Subjects in the intervention group will have free access to the 12-week internet-based self-help treatment. Participants are advised to spend at least 15 to 20 minutes per day on the program. If no module is finished over 4 weeks, this will be counted as a dropout for an additional sensitivity analysis. Patients in the control group will not receive any treatment or support from the researchers during the first 12 weeks after the initial survey. However, they are free to seek any other assistance they desire, including pharmacological and psychological treatments. All concurrent therapies will be repeat-measured using self-reports.

### Measures

Table 1 provides an overview of survey instruments at each time point, and more detailed descriptions are also provided below.

**Table 1:** Survey instruments at each time point.

Time point	Survey instrument
Pretreatment measurement (T1)	Video interview: Diagnostic Interview in Mental Disorders–Open Access (DIPS-OA). Primary outcomes (self-assessed via web-based questionnaire) Generalized anxiety disorder symptomatology (Generalized Anxiety Disorder–7; GAD-7) Quality of life (World Health Organization–Five Well-Being Test; WHO-5) Secondary outcomes (self-assessed via web-based questionnaire) Coping with difficulties in everyday life due to illness (Work and Social Adjustment Scale; WSAS) Work Capability (Institute for Medical Technology Assessment Productivity Cost Questionnaire; iPCQ) Health Literacy (Mental Health Literacy Scale; MHLS). Reduction of therapy-related expenses and burdens for patients and their relatives (Client Sociodemographic and Service Receipt Inventory; CSSRI) Secondary outcomes Demographic questionnaire Anxiety symptomatology (Beck Anxiety Inventory; BAI) Depressive symptoms (Patient Health Questionnaire–9; PHQ-9)
Intermediate measurement 6 weeks after T1 (T2)	Same as T1, except that there is no video interview Further surveys Negative effects (Negative Effects Questionnaire; NEQ) Short demographic questionnaire
Posttreatment measurement 12 weeks after T1 (T3)	Same as T2

### Video Interview

The DIPS-OA [26] is used to classify mental disorders and record inclusion and exclusion criteria. The person conducting the interview is part of the evaluation team (ie, part of the Faculty of Psychology and Sport Science, Giessen University) and independent of Selfapy.

### Primary Outcomes

Generalized anxiety disorder symptoms will be assessed with the Generalized Anxiety Disorder Scale–7 (GAD-7 [27]). The GAD-7 consists of 7 statements rated on a 4-point scale and has an internal consistency of α=.92.

The measurement used to assess well-being is the World Health Organization–Five Well-Being Test (WHO-5 [28]). The WHO-5 consists of 5 items rated on a 6-point Likert scale and has an internal consistency of α=.92.

### Secondary Outcomes

Coping with functional impairments will be assessed with the Work and Social Adjustment Scale (WSAS [29,30]), which consists of 5 items for which the true score is given as a number between 0 and 8. The WSAS is used to assess the degree of functional impairment in various domains. The WSAS has been used in comparable studies, such as a study by Gräfe et al [31]. The internal consistency of the WSAS ranges from α=.70 to α=.94 [30].

Work ability and productivity are assessed via the Institute for Medical Technology Assessment Productivity Cost Questionnaire (iPCQ [32]). The iPCQ asks about long-term (>2 weeks) and short-term (<2 weeks) absences from work. In addition, the iPCQ includes three questions on productivity losses as a result of illness-related work efficiency limitations. The iPCQ was validated by Friedli et al [33].

The Mental Health Literacy Scale (MHLS [34]) will be used to measure mental health literacy. The MHLS consists of 20 items, of which the first 15 items are rated on a 4-point Likert scale and the remaining items are rated on a 5-point Likert scale. The internal consistency of the overall scale is α=.87. The MHLS has previously only been validated in English and was translated into German for this study with the involvement of a natively bilingual collaborator in a translation-backtranslation process.

Therapy-related expenses and burdens for patients and their relatives are captured using the Client Sociodemographic and Services Receipt Interview (CSSRI [35]). The CSSRI asks participants to report the actual service utilization (eg, contact with health care providers, number of therapy sessions, amount of contact with psychotherapists and psychiatrists).

### Additional Outcomes

The Penn State Worry Questionnaire (PSWQ [36]) is used to assess worry as an accompanying syndrome of GAD; it consists of 16 items rated on a 5-point Likert scale. The PSWQ shows internal consistencies approximately in the range between α=.84 [37] and α=.86 [38] in GAD patients.

The Beck Anxiety Inventory (BAI [39]) is used to assess the severity of anxiety in adults and adolescents. It consists of 21 descriptive statements rated on a 4-point scale. The internal consistency of the BAI is α=.90 in clinical samples. The German version was validated by Margraf and Ehlers [40].

The Patient Health Questionnaire-9 (PHQ-9 [41]) is used to assess the presence and severity of depressive disorders. The PHQ-9 consists of 9 items rated on a 4-point Likert scale and has an internal consistency of α=.89.

To capture possible negative effects of the intervention, the Negative Effects Questionnaire (NEQ [42]) is used, which consists of 32 items rated on a 4-point Likert scale. The NEQ has an internal consistency of α=.95.

The adherence of the intervention group is recorded via the log files of the web-based platform Selfapy. The number of modules that have been worked on is recorded.

### Hypotheses

The main objective of this trial is to determine the efficacy of the 12-week Selfapy course for patients with GAD compared to a wait-list control group. Additional outcome data will be collected at 6 weeks. However, our confirmatory hypotheses, which will evaluate a positive health care effect, will focus on the 12-week outcomes. The following 2 hypotheses refer to our primary outcomes:

- GAD symptomatology (measured with the GAD-7 [27]) will significantly decrease with the use of the Selfapy course for GAD after 12 weeks compared to a wait-list control group.
- Perceived quality of life (measured with the WHO-5 [28]) will significantly increase after 12 weeks of the Selfapy course for GAD compared to a wait-list control group.

The following secondary outcomes will only be analyzed if the hypotheses for the primary outcomes are confirmed. They will also be tested using Bonferroni-Holm adjustment:

- Difficulties in daily life (measured with the WSAS [30]) will significantly decrease after using the Selfapy course for GAD after 12 weeks compared to a wait-list control group.
- Using the Selfapy course for GAD will lead to significantly better recovery of working ability (measured with the iPCQ [32]) after 12 weeks compared to a wait-list control group.
- Health literacy (measured with the MHLS [34]) will significantly improve with the use of the Selfapy course for GAD after 12 weeks compared to a wait-list control group.
- The extent of therapy-related efforts and burdens of patients and their relatives (measured with the CSSRI [35]) will be significantly reduced after using the Selfapy course for GAD compared to a wait-list control group after 12 weeks.

Our exploratory hypotheses do not address the target symptoms and are therefore not adjusted for α accumulation:

- Worry symptoms (measured with the PSWQ [36]) will significantly decrease with the use of the Selfapy course for GAD after 12 weeks compared to a wait-list control group.
- Self-rated anxiety symptoms (measured with the BAI [39]) will significantly decrease with the use of the Selfapy course for GAD after 12 weeks compared to a wait-list control group.
- Self-rated depressive symptoms (measured with the PHQ-9 [41]) will significantly decrease with the use of the Selfapy course for GAD after 12 weeks compared to a wait-list control group.
- The Selfapy course for GAD will not lead to any side effects compared to a wait-list control group after 12 weeks (measured with the NEQ [42]).

### Intervention

Selfapy is a program for the treatment of GAD [43]. The program uses evidence-based methods and exercises based on CBT and elements of mindfulness-based therapy (see, eg, Hoyer et al [44] and Volz and Stieglitz [45]). The web-based course consists of a core course, which includes mandatory and optional exercise content, and a subsequent set of modular specialization areas that are individually selectable (Table 2). The program can be used via desktop browsers and mobile devices. The web-based course is divided into different lessons, each covering a specific topic, such as exposure, mindfulness, or problem-solving training, and consists of informative texts, videos, audio, and interactive exercises. Table 2 provides an overview of the core course with examples and possible in-depth areas and gives a brief overview of their content.

**Table 2:** Overview of modules with example content of the generalized anxiety disorder program.

Module		Content
Your start		In the first module, users can describe their problems and set personal goals.
First findings		This module focuses on psychoeducation. Users learn to recognize and understand the background of their problems. In addition, users begin keeping a worry log to identify triggers and patterns regarding their worries and fears.
The mental level		This module deals with the thoughts behind the users’ anxiety disorder. With the help of various exercises, users learn to question their worries and replace them with more realistic thoughts.
The physical level		In this module, users learn about the fear response and the physical symptoms of generalized anxiety disorder.
The behavioral level		In this module, users learn about how avoidance and reassurance contribute to maintaining their anxiety disorder and how the 3 levels of anxiety interact.
The first exposure		In this module, users are introduced to exposure-based methods. They learn how exposure works and why it is helpful in the treatment of anxiety disorders. Users will also receive information on how to perform an in sensu exposure and consider when to do so.
The second exposure		This lesson is dedicated to in vivo exposure. Users are informed about what it looks like and what to consider when performing it.
**Optional areas of specialization**		
	Self-efficacy	In this module, users learn about self-efficacy. Through various exercises, they are trained in self-efficacy so as to believe more in themselves and their abilities.
	Mindfulness	A mindful approach to life can have a supportive effect to better deal with one’s problems. With the help of formal and informal mindfulness exercises, users learn how to integrate a mindful approach to themselves into everyday life.
	Social environment	This module deals with the impact of the social environment on one’s own life. Through social networks and communication exercises, users can optimize their social support and strengthen their social skills.
	Problem solving training	This module provides training in problem-solving skills. Users learn to perceive a concrete problem, grasp reaction possibilities, and implement an action to change the situation.
	Your anti-anxiety package	In the final module, users take stock of the completed course. They summarize which content was particularly helpful and where they can still improve their anxiety disorder. At the end of the program, the user gains an antianxiety package to use when problems arise again or a relapse has already set in.

### Randomization and Blinding

Subjects meeting the inclusion criteria will be randomly assigned to 1 of 2 groups and will receive either (1) immediate access to the minimally-guided Selfapy GAD course (the intervention group) or (2) access to the Selfapy GAD course after a waiting period of 12 weeks (the control group).

Randomization takes place after the participant has finished the entry questionnaire, which is conducted by a member of the psychology department who is not involved in the project, using a computer-assisted procedure. Random assignment is done only if participants fulfill the inclusion criteria for the study. Until this time, which group the person will be assigned to if included is unknown (allocation sequence concealment). Participants will be assigned to 1 of the 2 groups in a nonstratified 1:1 ratio. Subjects will be informed of the outcome of the random assignment via email. Participants are told that the waiting time will randomly vary between 0 and 12 weeks. Thus, patients in the control group will not know that the sample is divided into 2 groups. One group will start the intervention immediately while the other group will wait 12 weeks before receiving access to the therapy. The diagnostic interviewers will be blind to the group membership of the participants. After completing data collection, statistical analysis of the outcomes will also be performed in a blinded manner.

### Power and Sample Size

The between-group effect size estimate is based on recent meta-analytic evidence for effect sizes in unguided web-based psychological interventions for anxiety disorders (Cohen *d*=0.45; see, for example, McCall et al [46]). Even though Selfapy is minimally guided, we chose this meta-analysis since the degree of guidance is not associated with the effect size [16,17]. This effect size will be used as the basis for sample size design. For the planned mixed model with 2 measurement time points with a general correlation structure [47], a directed hypothesis, a group allocation of 1:1, a power of 0.80, and an α level of .025 after Bonferroni-Holm correction, a total of 156 patients (n=78 per group) are needed. The number of cases was calculated using the R tool *longpower* [48]. For the secondary outcomes, this sample size yields a power of 1–β=.67.

### Statistical Analyses

For the psychometric outcome analyses, all patients who were randomly assigned to the 2 conditions and completed the initial survey (T1) will be included in an intention-to-treat (ITT) analysis. All available data will be used for this purpose. Missing values in the data will be replaced by multiple imputations (multivariate imputation by chained equations with n=5 imputations) based on the control arm, using the variables “age” and “gender” as predictors in addition to the measurement-repeated stress indicators. In addition, last-observation-carried-forward, baseline-observation-carried- forward, reference-based-multiple imputation (jump-to-reference approach [49]), and completer sensitivity analyses will be performed.

The confirmatory analysis of the primary outcomes consists of calculating a mixed model with 2 measurement time points and a general correlation structure [47]. A random effect for the subjects is calculated (random intercept) and 3 fixed effects (group assignment, time, and the interaction of the 2 predictors). The 2 measurement time points are nested within subjects. The primary outcomes will be evaluated using a Bonferroni-Holm correction for α error inflation. Secondary confirmatory outcomes will only be analyzed if the results of the primary analysis confirm the hypotheses, and the same mixed model with a random intercept for the subject will be applied. Again, a Bonferroni-Holm correction will be used.

Independent 2-tailed *t* tests and chi-squared tests will be used to estimate differences between groups in pretreatment sample characteristics. In addition to the ITT sample, a “per-protocol” sample sensitivity analysis is defined for exploratory analyses, including all patients in the intervention group who completed at least 4 of the modules.

There are different approaches to calculating effect sizes for mixed modeling data in the literature [50]. Hedges [50] and Westfall et al [51] propose an effect size based on the Cohen *d*, which is also used for power analysis [47]:




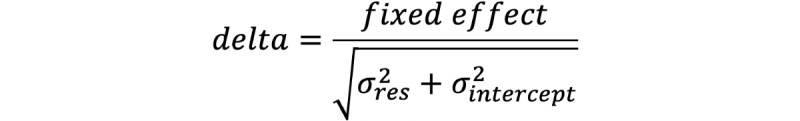




To assess the magnitude of the treatment effects, the fixed interaction effect of time and group assignment is divided by the root of the summed variances of the random effects. Effect sizes can be roughly interpreted according to the Cohen *d*: effect sizes of 0.2 are considered small, 0.5 moderate, and 0.8 large [52]. Differences in response rates and additional health care service utilization rates are examined with 2-tailed *t* tests and chi-squared tests.

All data analyses are performed without knowing group membership (blinded data analysis). The evaluator does not know which expression of the group variable indicates membership in the intervention group and which indicates membership in the control group. Multimedia Appendix 1 shows example code for the R analysis of the outcomes.

### Ethical Considerations

Participants are asked about suicidality at all measurement time points (T1, T2, and T3). For safety reasons, subjects will be contacted if they report suicidal thoughts and an emergency plan is drawn up with them. Nevertheless, the subject’s data will not be passed on to the police or other authorities. If participants endorse suicidality, they will be excluded from further data analyses, and only the assessments up to the occurrence of suicidality will be used.

The study center at the University of Giessen is responsible for storing and analyzing patient data. Besides the initial interview, all data are collected via a web-based platform (SoSci Survey; SoSci Survey GmbH) that uses SSL encoding to protect the data and stores them on servers located in Germany. Participant data are stored pseudonymously in order to access contact data in cases of suicidality. However, after the study is finished, the data will be immediately deidentified. Written informed consent is obtained from all participants before participation, and the ethics committee of the Faculty of Behavioral and Empirical Cultural Science at the study center at Heidelberg University approved the clinical protocol (AZ Prüß 2021 1/1). The trial is registered at the German Clinical Trials Register (DRKS00023800) and follows the ethical principles of the Declaration of Helsinki. All patients receive an allowance of €30 (US $33) after completing the study.

## Results

By May 2023, all participants completed the study, and a paper reporting the results is being prepared. The report will be submitted for publication in the coming months.

## Discussion

This study tests the efficacy of a minimally guided web-based intervention (Selfapy) for the treatment of GAD in routine clinical care. The study has several strengths. First, the study was designed to strike a balance between a high degree of internal validity (eg, randomization, a standardized diagnostic procedure), allowing us to attribute observed group differences to the intervention with sufficient certainty and a high external validity (eg, allowing participants to use additional health care services) to evaluate the effects in an ecologically valid context. In particular, we did not prepare artificial measures to increase adherence to gain a representative impression of routine care patient adherence. Second, to the best of our knowledge, this study is one of the most highly powered studies on iCBT for GAD [13]. Third, we will investigate outcomes beyond symptom reduction, such as functioning and health care use. Limitations of this study include the lack of an active control group instead of a wait-list control group, even though this design allows only limited conclusions regarding the specificity of the effects found. We chose this design in an attempt to assess the incremental effect of Selfapy for GAD patients compared to the current situation in the German health care system. Patients who want to undergo outpatient psychotherapy in Germany have to wait for an average of 20 weeks to start treatment [19]. One of the main advantages of an internet intervention like the one investigated in this study is its availability. If implemented in routine clinical care, patients can make use of these interventions without delay and can expect results comparable to traditional face-to-face therapy [14]. One could argue that the waiting period in the control group (12 weeks) is too short, given the average waiting time in the health care system of 20 weeks. Still, we decided to restrict the waiting time in the control group to 12 weeks for several reasons. First, for ethical reasons, we wanted to limit the waiting time as much as possible. Withholding a likely helpful intervention should only be done as long as necessary. Again, we want to highlight that patients in the control group, as well as those in the intervention group, were allowed to seek additional treatment and help if they thought it necessary. No patients were asked to refrain from any other mental health services. Second, 12 weeks is the time the intervention takes when completed on the recommended schedule. Therefore, fixing the control group to the same measurement schedule as the intervention allows us to evaluate the effects of having access to Selfapy for GAD compared to not having access to this intervention. Another limitation lies in the use of deception in telling the patients that the intervention will start some time between 0 and 12 weeks later. This is done to attenuate dissatisfaction with being in the control group. However, patients in the control group may still feel dissatisfied and unlucky when they have to wait what they think is the maximum amount of time for the intervention. Also, we do not assess the frequency of personality disorders, which may be reasonable because GAD is associated with higher rates of personality disorders [53]. Further limitations include the lack of follow-up measurement, the lack of a power calculation for the secondary outcomes, and the lack of a mindfulness measure despite the intervention including a mindfulness-based approach.

Due to current technological progress, the implementation of web-based interventions may be an important addition to the German health care system. This may reduce barriers to treatment provision and complement current clinical care. Therefore, we aim to assess a routine-care web-based intervention for its impact on symptom reduction, well-being, work capability, and other measures of disease burden.

## Appendix

Multimedia Appendix 1 Example code for the analysis in R.

## Abbreviations

BAI: Beck Anxiety Inventory
CBT: cognitive behavioral therapy
CSSRI: Client Sociodemographic and Service Receipt Inventory
DIPS-OA: Diagnostic Interview in Mental Disorders-Open Access
GAD-7: Generalized Anxiety Disorder 7
iCBT: internet-delivered cognitive behavioral therapy
iPCQ: Institute for Medical Technology Assessment Productivity Cost Questionnaire
ITT: intention-to-treat
MHLS: Mental Health Literacy Scale
NEQ: Negative Effects Questionnaire
PHQ-9: Patient Health Questionnaire 9
WHO-5: World Health Organization–Five Well-Being Test
WSAS: Work and Social Adjustment Scale

## Data Availability

The data sets generated during the upcoming study will be available in the OSF repository [54].
