# Supplementary material for: Evaluation of a Web-Based Self-Help Intervention for Patients With Generalized Anxiety Disorder: Protocol for a Randomized Controlled Trial
Source: JMIR Res Protoc. 2023 Jul 26;12:e41440. doi: 10.2196/41440 (PMC10413245; doi:10.2196/41440)
Supplement: Multimedia Appendix 1 [file resprot_v12i1e41440_app1.docx]

library(mlmi)

library(bootImpute)

library(lme4)

library(tidyverse)

library(plyr)

library(mice)

library(broom.mixed)

library(miceadds)

library(zoo)

library(merTools)

library(Amelia)

#df is the Outcome-dataframe with the variables ID (id), dummy-#variable for the treatment (trt) and three measurements with #the according time points (y0, y1, y2), age (age), and sex (sex)

set.seed(1234)

# MICE-Imputation ----

# Imputation building on control group

ignore_vector <- df$trt

ignore_vector <- as.logical(ignore_vector)

#MICE-Imputation, 5 imputations

imp_mice <- mice(data = df, m = 5, ignore = ignore_vector)

#transform mids-object in a list

imp.data <- as.list(1:5)

for(i in 1:5){

  imp.data[[i]] <- complete(imp_mice, action=i) }

#transform into long-format

imp.data_long <- lapply(imp.data, pivot_longer, cols = 3:6, names_to = "time", values_to = "value")

#Model calculation for each list element

model_mice <- lmerModList(value ~ trt*time + (1|id), data = imp.data_long)

#Random and Fixed Effects

modelRandEffStats(model_mice)

modelFixedEff(model_mice)

# LOCF-imputation -----

#Transfomr into long format

df_long<- pivot_longer(df, cols = 3:5, names_to = "time", values_to = "value")

df_long_locf <- df_long

df_long_locf$value <- na.locf(df_long$value) #LOCF-Imputation

#model calculation

mod.locf <- lmer(value ~ trt*time + (1|id), df_long_locf)

summary(mod.locf) # Random and Fixed effects

#BOCF-imputation -----

df_bocf <- df

df_bocf$y1 <- ifelse(is.na(df$y1), df$y0, df$y1) #BOCF für y1

df_bocf$y2 <- ifelse(is.na(df$y2), df$y0, df$y2) #BOCF für y2

#transform into long-format

df_long_bocf<- pivot_longer(df_bocf, cols = 3:5, names_to = "time", values_to = "value")

mod.bocf <- lmer(value ~ trt*time + (1|id), df_long_bocf)

summary(mod.bocf) # Random and Fixed effects

#Multiple imputations according to J2R-approach

j_to_r.wide <- bootImpute(df, refBasedCts, nBoot=500, nImp=2,

outcomeVarStem="y", nVisits=2, trtVar="trt",baselineVars="y0", type="J2R", M=2)

# Transform into long format

j_to_r.long <- lapply(j_to_r.wide, pivot_longer, cols = 3:5, names_to = "time", values_to = "value")

#Model calculation

model_jtor <- lmerModList(value ~ trt*time + (1|id), data = j_to_r.long)

#Random Effects:

modelRandEffStats(model_jtor)

#Fixed Effects:

modelFixedEff(model_jtor)
